# Supplementary material for: A Convenient and Highly Efficient Strategy for Esterification of Poly (γ-Glutamic Acid) with Alkyl Halides at Room Temperature
Source: Polymers (Basel). 2024 Dec 25;17(1):10. doi: 10.3390/polym17010010 (PMC11723209; doi:10.3390/polym17010010)
Supplement: Supplementary file 1 [file polymers-17-00010-s001.zip › polymers-3363553-supplementary.pdf]

Electronic supplementary information

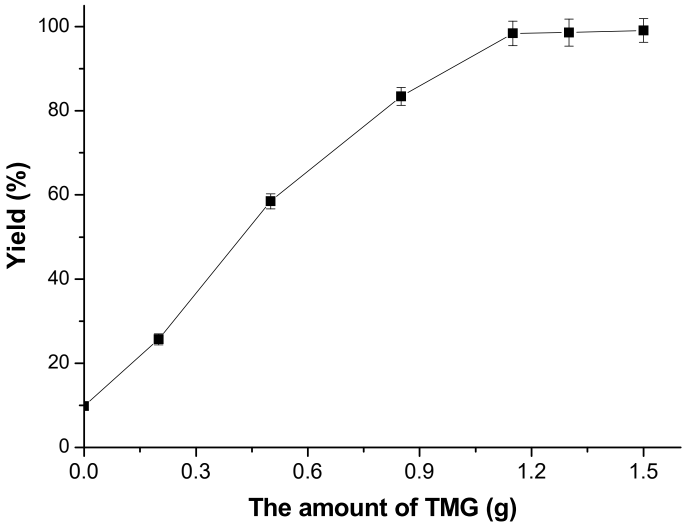

Figure S1. Effect of the amount of TMG added on reaction process. Reaction conditions: γ-PGA-Na (768 mg), propyl bromide (3.69 g), NMP (50 mL), reaction time (3 h). The yield of esterification was determined by gravimetric method.

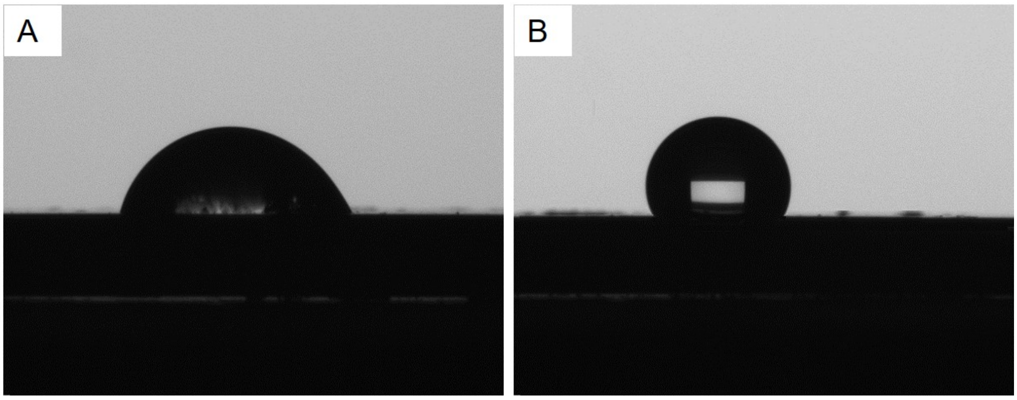

Figure S2. Contact angles of water on γ-PGA-Na (A) and γ-PGA propyl ester (B). (A) CA= 63.2º and (B) CA=118.7º.

Table S1

Esterification of γ-PGA-Na with various *n*-alkyl bromide homologues using TMG as a promoter in NMP at room temperature [a]

Entry	*n*-alkyl bromide	Time /h	Yield /%
1	Ethyl bromide	3	97.1±1.21 [b]
2	*n*-Propyl bromide	3	98.6±0.72
3	*n*-Butyl bromide	3.5	95.5±1.30
4	*n*-Hexyl bromide	3.5	92.3±1.52
5	*n*-Octyl bromide	4	91.8±0.82
6	*n*-Decyl bromide	4.5	89.5±1.64

[a] Reaction conditions: γ-PGA-Na (768 mg), *n*-alkyl bromide homologues (0.03 M), NMP (50 mL) and TMG (1.15 g) as a promoter. The yield of esterification was determined by gravimetric method.

[b] Relative standard deviation.
